# Sump syndrome following hepaticojejunostomy: a case report of IgG4-related sclerosing cholangitis

**DOI:** 10.3389/fmed.2025.1730125

**Published:** 2026-01-12

**Authors:** Liqin Ruan, Weili Chen, HanYing Mei

**Affiliations:** 1 Department of Hepatobiliary Surgery,Jiujiang City Key Laboratory of Cell Therapy, Jiujiang No. 1 People’s Hospital, Jiujiang, Jiangxi, China; 2 Laboratory of Rheumatology and Immunology Department, Jiujiang City Key Laboratory of Cell Therapy, Jiujiang No. 1 People’s Hospital, Jiujiang, Jiangxi, China

**Keywords:** cholangitis, glucocorticoids, IgG4-related sclerosing cholangitis, Roux-en-Y hepaticojejunostomy, sump syndrome

## Abstract

This report presents a case of IgG4-related sclerosing cholangitis (IgG4-SC) in a patient who ultimately died from a complex postoperative infection. The patient was a 59-year-old male who underwent resection of a hilar lesion and Roux-en-Y hepaticojejunostomy (RYHJ) for obstructive jaundice 7 years ago, with postoperative pathology confirming IgG4-SC. However, from 4 months after surgery, the patient experienced recurrent fever episodes that persisted for 7 years. The clinical presentation was consistent with sump syndrome. Both recurrent infections and IgG4-SC disease activity can elevate IgG4 levels, complicating clinical assessment. Despite aggressive antibiotic therapy combined with immunosuppressive treatment, the infections recurred. The patient eventually succumbed to sepsis in August 2025, triggered by recurrent cholangitis. This case underscores the importance of differentiating IgG4-SC from hilar cholangiocarcinoma (HC) to avoid unnecessary surgery. Moreover, it highlights that in post-hepaticojejunostomy patients, distinguishing whether elevated IgG4 levels signify disease activity or are secondary to infection is essential for guiding therapy.

## 1 Introduction

IgG4-related disease (IgG4-RD) is a systemic immune-mediated disorder with incompletely understood etiology, potentially associated with genetic predisposition, environmental factors, and autoimmune dysregulation. IgG4-RD is characterized by a diffuse or mass forming inflammatory reaction rich in IgG4-positive plasma cells associated with fibrosclerosis and obliterative phlebitis (1). IgG4-SC, a biliary manifestation of IgG4-RD, primarily involves inflammation and fibrosis of the bile ducts, leading to stenosis or obstruction (2). The imaging findings and clinical presentation of IgG4-SC closely resemble those of HC, posing diagnostic challenges. This report presents a case of IgG4-SC initially misdiagnosed and subsequently diagnosed by pathology. In patients with IgG4-SC, elevated IgG4 levels accompanied by fever should prompt consideration of secondary infection rather than primary disease activity. In cases of recurrent cholangitis following hepaticojejunostomy, surgical intervention or endoscopic placement of a Duckbill-type antireflux self-expandable metal stent may be warranted to address the underlying cause and prevent further episodes.

## 2 Case presentation

A 59-year-old male patient has experienced recurrent fever leading to repeated hospitalizations since undergoing a hepaticojejunostomy 7 years ago. In April 2018, the patient underwent resection of hilar bile duct lesions and RYHJ at Beijing Hospital for obstructive jaundice. The operative report indicated that end-to-side anastomoses were performed separately between the right anterior, right posterior, and left hepatic ducts and the jejunum to construct a Roux-en-Y anastomosis, with a jejunal limb (50 cm). Postoperative pathology revealed bile duct tissue (hilar bile duct mass) with extensive infiltration of IgG4-positive plasma cells (>10/HPF), accompanied by significant fibrosis leading to bile duct wall thickening. This finding confirmed a diagnosis of IgG4-SC. After surgery, the patient was administered low-dose corticosteroid therapy (30 mg per day) for 3 months before discontinuing treatment without medical advice.

**Figure 1:**
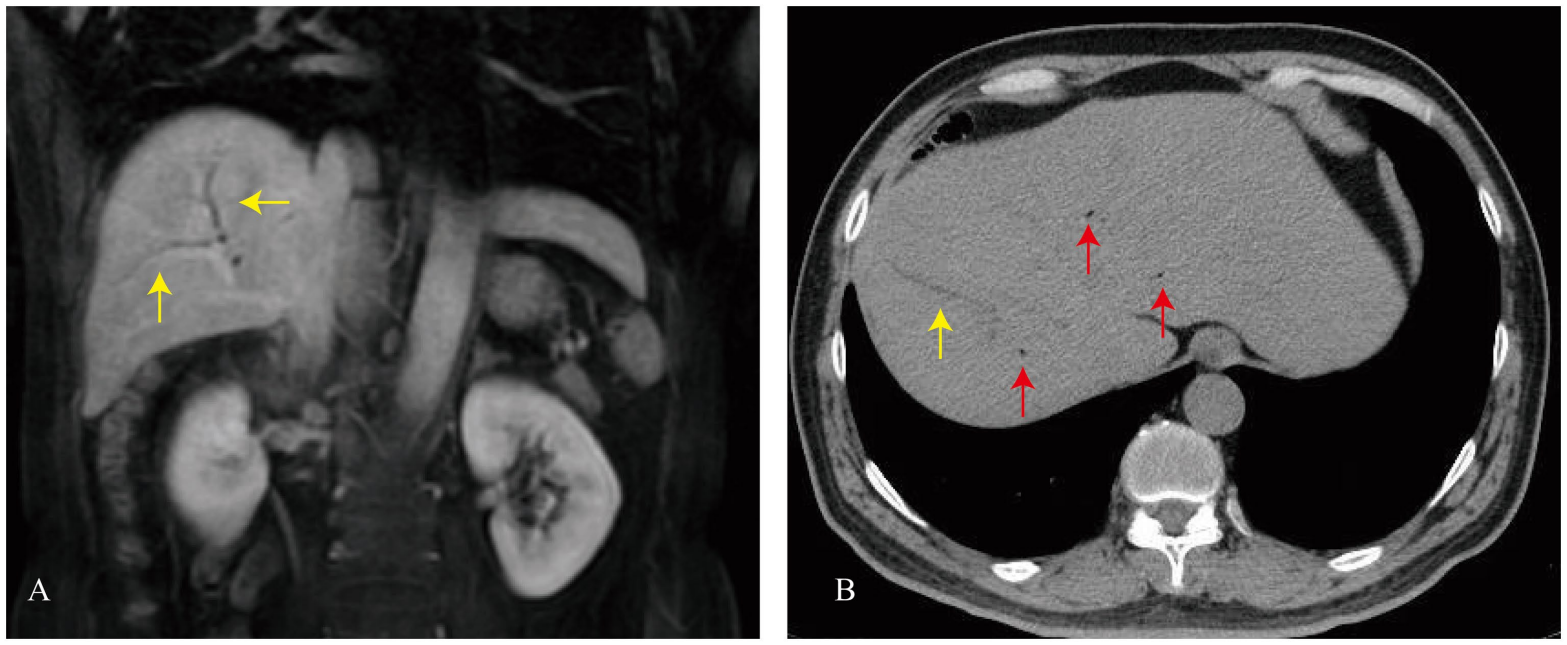
**(A)** MRI of the abdomen showing mild intrahepatic bile duct dilation (yellow arrow). **(B)** CT of the abdomen showing pneumobilia (red arrow) and mild intrahepatic bile duct dilation (yellow arrow).

Four months postoperatively (August 2018), the patient began experiencing fever accompanied by pain in the back and body. To further elucidate the etiology, a comprehensive systemic examination was performed, including transesophageal echocardiography and contrast-enhanced computed tomography (CT) of the entire abdomen; however, no definitive source of infection was identified. Antibiotic therapy was effective in normalizing body temperature. Three years after surgery, the patient came to our department for treatment. Laboratory investigations revealed an elevated white blood cell count (WBC) of 12.03 × 10^9^/L (reference range 3.6–10 × 10^9^/L), a C-reactive protein (CRP) level of 16.94 mg/L (0–5 mg/L), and an erythrocyte sedimentation rate (ESR) of 90 mm/h. Alanine aminotransferase (ALT) was 39.2 U/L (reference range 0–49 U/L), and aspartate aminotransferase (AST) was 24.1 U/L (reference range 0–40 U/L). In contrast, alkaline phosphatase (ALP) was elevated at 297 U/L (reference range 40–150 U/L), and γ-glutamyl transferase (γ-GT) was markedly elevated at 395 U/L (reference range 0–50 U/L). MRI/MRCP was performed, which showed mild intrahepatic bile duct dilation and no evidence of biliary-enteric anastomotic stenosis (Figure 1A). Five years after surgery, an abdominal CT scan demonstrated mild intrahepatic bile duct dilation and scattered pneumobilia (Figure 1B). In September 2023, a markedly elevated serum IgG4 level (7.066 g/L) was observed, suggesting the possibility of active IgG4-RD. Based on consultation with the rheumatology department, the patient was started on a combination therapy of methotrexate and triamcinolone acetonide and antibiotic therapy. On October 27, 2023, follow-up testing showed a serum IgG4 level of 2.834 g/L. However, recurrent fever and back pain persisted, with repeated blood cultures detecting *Escherichia coli* and *Enterococcus faecium* infections. Piperacillin therapy proved ineffective, leading to a switch to meropenem for fever control. Due to the lack of improvement in fever symptoms despite oral corticosteroids and immunosuppressants, methotrexate and triamcinolone were discontinued. Following cessation, the patient’s IgG4 levels rose again to 5.335 g/L, prompting the resumption of oral corticosteroids and immunosuppressants, which subsequently reduced IgG4 levels to 2.655 g/L.

In July 2024, an external CT review revealed multiple small calculi in the intrahepatic bile ducts, scattered areas of pneumobilia, mild bile duct dilation, and multiple duodenal diverticula. In November 2024, the patient stopped corticosteroid and immunosuppressant therapy again due to concerns about potential adverse effects.

**Figure 2:**
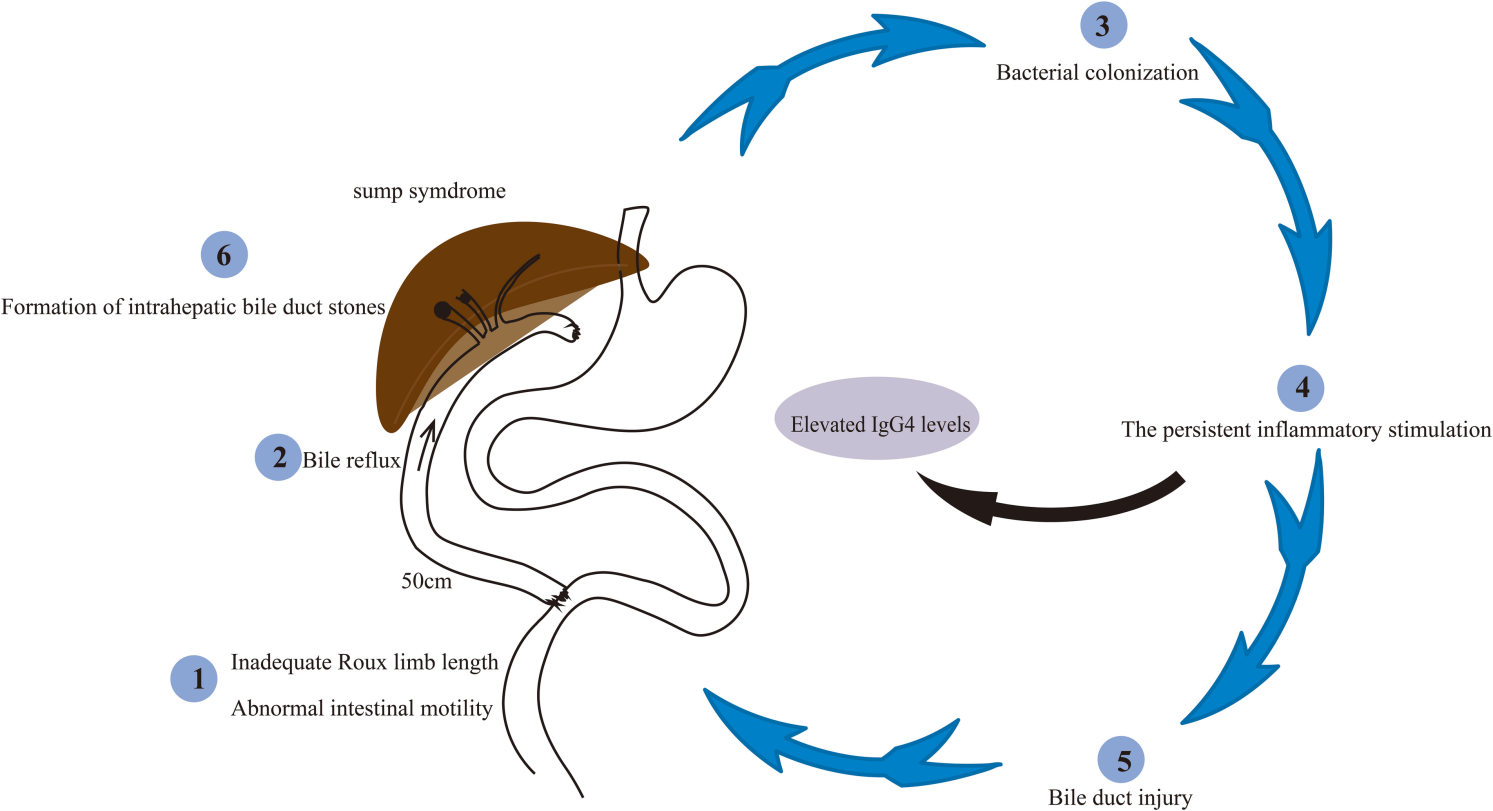
Pathogenesis of recurrent cholangitis (sump syndrome) following RYHJ. An inadequate Roux limb length (50 cm) initiates bile reflux, bacterial colonization, chronic inflammation, and biliary injury, leading to formation of intrahepatic duct stone. Persistent inflammation contributes to elevated serum IgG4 levels.

The patient had planned to undergo surgery at a tertiary hospital; however, after careful deliberation, the patient opted for continued conservative treatment (rotation of antibiotics), considering concerns over the uncertainty of preventing recurrent fever postoperatively. In May 2025, serum IgG4 levels surged to 10.32 g/L. From 4 months after the hepaticojejunostomy, the patient has suffered from recurrent cholangitis, accumulating over 80 hospitalizations to date. Although antibiotic therapy alleviated the febrile symptoms, the frequency of fever episodes remained approximately once per month. The patient ultimately succumbed to sepsis in August 2025. The timeline of the disease course and the dynamic changes in IgG4 levels are illustrated in Figure 2.

## 3 Discussion

IgG4-RD is a systemic immune-mediated disorder with incompletely elucidated etiology, potentially associated with genetic predisposition, environmental factors, and autoimmune responses, initially identified in autoimmune pancreatitis (AIP) (1). IgG4-SC represents a biliary manifestation of IgG4-RD and ranks as the second most common subtype. The hallmark pathological features of IgG4-SC include dense periductal infiltration of IgG4-positive plasma cells and lymphocytes, followed by progressive fibrosis and biliary strictures (3). Approximately 88% of IgG4-SC cases coexist with AIP (4). Clinically, IgG4-SC primarily manifests as abdominal pain, jaundice, and pruritus, with some patients developing acute cholangitis, weight loss, or diabetes (2). IgG4-SC without concurrent AIP is defined as isolated IgG4-SC. Isolated IgG4-SC is relatively rare in clinical practice and often mimics cholangiocarcinoma (CCA), as both conditions exhibit similar imaging findings of hilar biliary strictures, thereby posing significant diagnostic challenges (5–8).

Distinguishing IgG4-SC from CCA is a critical clinical challenge. Approximately 15% of patients undergoing major surgery for suspected biliary or pancreatic malignancies are ultimately diagnosed with IgG4-SD, underscoring the importance of accurate preoperative differentiation. Serum IgG4 levels exceed 1.4 g/L in 75–80% of patients with IgG4-SC, and concentrations greater than 5.6 g/L (four times the upper limit of normal, ULN) strongly support the diagnosis. Although 10–15% of CCA and pancreatic cancer patients also exhibit elevated IgG4, their levels typically remain below 5.6 g/L. Imaging features of IgG4-SC are classified into four types: Type 1 presents with isolated distal bile duct stenosis, requiring differentiation from pancreatic cancer and CCA; Type 2 involves stenosis in both intrahepatic and extrahepatic bile ducts, which must be distinguished from primary sclerosing cholangitis (PSC). Type 3 and Type 4, characterized by hilar hepatic lesions with or without distal bile duct stenosis, necessitate careful differentiation from CCA using ERCP-guided bile duct biopsy and intraductal ultrasonography.

Corticosteroids serve as the first-line therapy for IgG4-SC (1, 9–11), with an initial regimen of prednisolone at 0.5–0.6 mg/kg/day (30–40 mg/day) for 4 weeks, followed by a taper of 5 mg every 2 weeks until a maintenance dose of ≤10 mg/day is reached (12). Immunomodulatory agents such as azathioprine or methotrexate are frequently introduced to minimize corticosteroid exposure and lower the risk of relapse, particularly in patients with a history of recurrence or a high risk of recurrence (13). Rituximab is recommended as a second-line treatment for patients who respond poorly to conventional therapy, experience relapse, or exhibit corticosteroid intolerance, demonstrating significant efficacy in alleviating symptoms and reducing serum IgG4 levels (11).

The management of hilar cholangiocarcinoma (HC) predominantly relies on surgical resection, with radical resection combined with hepaticojejunostomy being the standard approach (14). In the present case, the patient was initially misdiagnosed with HC due to obstructive jaundice and imaging findings of irregular wall thickening (20 × 16 mm) in the upper common bile duct, prompting hilar lesion resection and hepaticojejunostomy. Short-term complications of RYHJ include bile leakage, whereas long-term complications often involve anastomotic strictures (15). Recurrent cholangitis following RYHJ is commonly attributed to postoperative anastomotic strictures (15). However, studies have identified sump syndrome as a rare cause of recurrent cholangitis post-RYHJ (16). Classically, sump syndrome refers to a rare long-term complication of choledochoduodenostomy (CDD), typically occurring in the absence of anastomotic stenosis (17). Although uncommon, it is a potentially life-threatening complication. It arises from debris accumulation (e.g., calculi, food residues, or pus) within the distal bile duct stump, leading to recurrent cholangitis or pancreatitis, with an occurrence rate of 0–15.7% (18). With advancements in endoscopic techniques, CDD has declined in clinical use. In 1996, Morrissey et al. (16) first reported sump syndrome following RYHJ, characterized by fever, abdominal pain, and abnormal liver function. Marangoni et al. (19) described six cases of post-RYHJ sump syndrome, with initial cholangitis episodes occurring 7 months to 36 years postoperatively, requiring 1–9 hospitalizations, these cases exhibited no anastomotic strictures.

Sump syndrome mechanisms linked to an inadequate Roux limb length and abnormal intestinal motility, which challenges the conventional belief that a 50-cm limb is sufficient for reflux prevention (19, 20). Another study suggested that dysmotility in the jejunal limb distal to the anastomosis may promote bacterial overgrowth, precipitating acute cholangitis (20). Furthermore, the remnant common bile duct with post-hepaticojejunostomy, may act as a “sump” for bacterial/debris accumulation due to impaired drainage (21).

The patient experienced recurrent postoperative fever with elevated serum IgG4 levels. However, no definite source of infection was initially identified. While reactivation of IgG4-SC was suspected and treatment with glucocorticoids and immunosuppressants was initiated, the patient’s fever persisted intermittently despite a decrease in IgG4 levels. This clinical course aligns with the exclusion criteria of “The 2019 American College of Rheumatology/European League Against Rheumatism classification criteria for IgG4-related disease” (22). Specifically, the patient exhibited documented recurrent fevers (>38 °C) over 7 years with clear evidence of bacterial infection, which contradicts the presentation of primary IgG4-RD. Furthermore, the patient demonstrated no objective clinical response to high-dose glucocorticoids (prednisone ≥40 mg/day), another critical exclusion criterion. The criteria explicitly states that improvement in serum IgG4 alone, without clinical or radiological improvement, does not constitute a response (22). Literature review further indicates that IgG4-related cholangitis is rarely associated with fever. A large-scale Japanese study reported that only 10% of patients presented with cholangitis symptoms, primarily characterized by jaundice and abdominal pain (10). Although elevated serum IgG4 is a hallmark of IgG4-RD, it is non-specific and can occur in infections (23). Therefore, we hypothesize that the elevated IgG4 was a pseudo-elevation resulting from chronic immune stimulation due to recurrent *E. coli* infection rather than disease recurrence. This is consistent with findings that significant IgG4-positive plasma cell infiltration can occur in resected bile duct tissues of sump syndrome patients following RYHJ (24).

**Figure 3:**
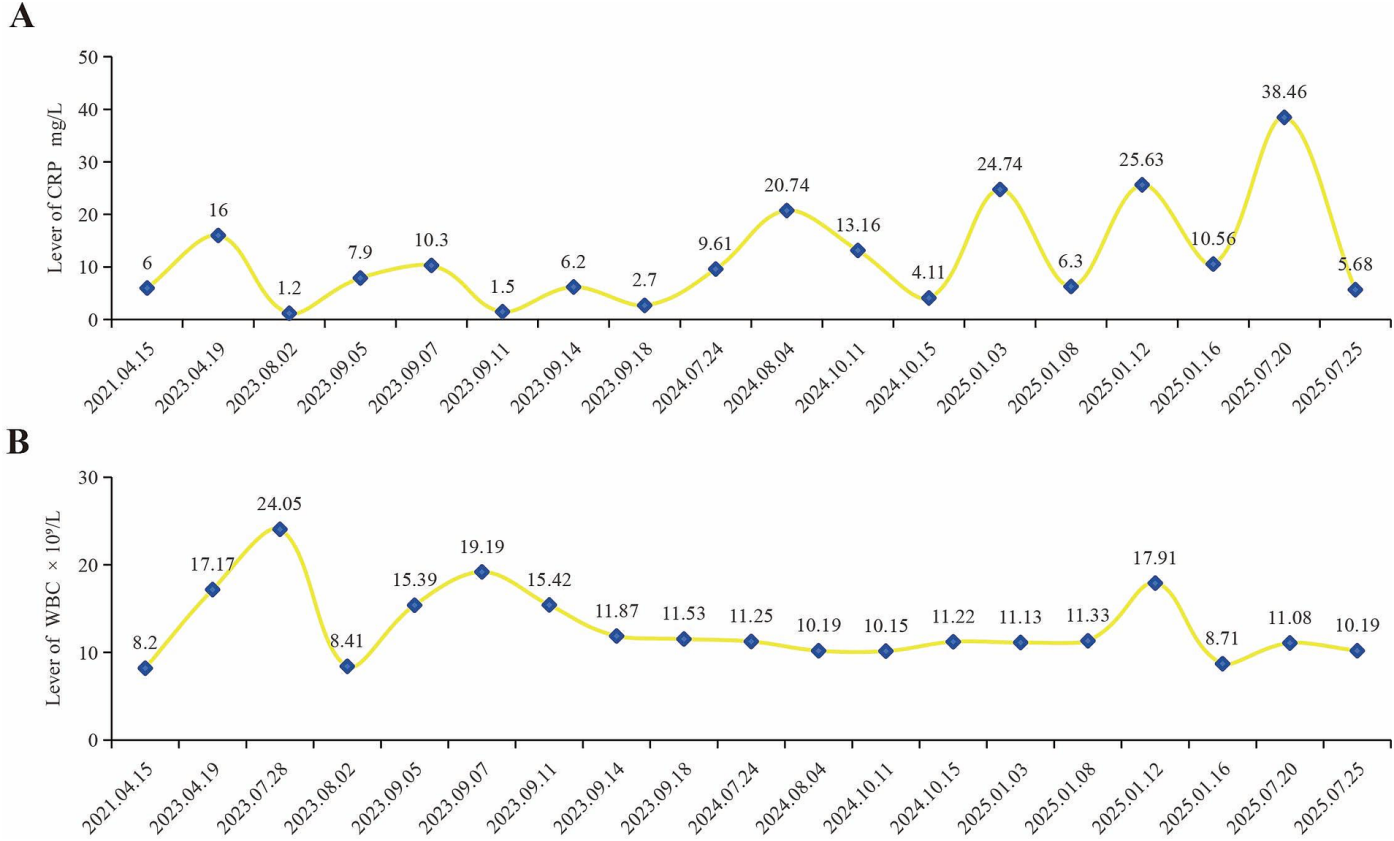
The dynamic changes in inflammatory parameters (CRP **(A)** and WBC **(B)**) in our hospital.

**Figure 4:**
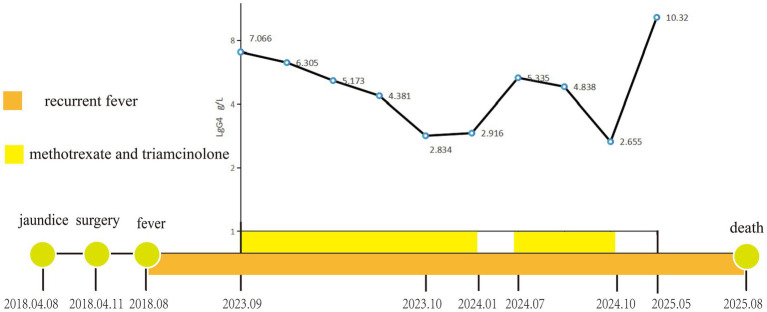
Timeline of serum IgG4 levels and key clinical events.

Sump syndrome is defined as bile stasis and reflux of choledochoenteric contents into the common bile duct and cholangitis caused by the siphon effect after choledochoduodenostomy or hepatoenterostomy, without evidence of anastomotic stricture. In this case, the diagnosis of sump syndrome was established clinically based on clinical presentation, imaging findings, and the process of exclusion. The patient presented with recurrent episodes of fever following RYHJ that could not be explained by other etiologies. Laboratory investigations revealed elevated WBC and CRP levels (Figure 3), along with positive blood cultures. MRI/ MRCP and CT scans demonstrated pneumobilia and the absence of stenosis at the hepaticojejunostomy site, thereby excluding the most common cause of recurrent cholangitis after RYHJ and aligning with the characteristics of sump syndrome. Comprehensive evaluations, including transesophageal echocardiography and contrast-enhanced CT of the entire abdomen, were conducted to rule out other potential infectious foci, such as endocarditis or pneumonia, which could account for the prolonged, recurrent fever. These findings strongly suggested a diagnosis of sump syndrome. To confirm the diagnosis of sump syndrome, specialized investigations that directly demonstrate biliary stasis or enteric reflux are superior, such as hepatobiliary iminodiacetic acid (HIDA) scan, 99mTc-N-pyridoxyl-5-methyltryptophan (99mTc-PMT) hepatobiliary scintigraphy, percutaneous transhepatic cholangiogram (PTC), endoscopy, barium meal or oral contrast studies (19, 25–28). These modalities can provide definitive evidence of bile stasis at the anastomosis site. However, a limitation of this case is the lack of direct evidence from gastrointestinal contrast studies, endoscopic retrograde cholangiopancreatography (ERCP) or surgery. Additionally, the patient developed intrahepatic bile duct stones during the later course. The mechanism may involve repeated biliary reflux leading to secondary bacterial colonization, which induces persistent inflammatory stimulation, resulting in bile duct injury and ultimately promoting the formation of intrahepatic bile duct stones (Figure 4).

Currently, no standardized treatment protocol exists for sump syndrome. Previous literature suggests that some patients may achieve resolution of acute cholangitis secondary to sump syndrome through revisional surgery. This involves elongation of the jejunal limb to 70–100 cm. However, mild cholangitis may persist in a minority of cases. For refractory cases, lifelong antibiotic rotation may be required (19, 25). Notably, two Japanese case reports demonstrated that placement of duckbill-type anti-reflux self-expandable metal stents (AR-SEMS) at the anastomotic site could effectively manage reflux cholangitis caused by non-obstructive afferent loop syndrome (27, 28). Sump syndrome following hepaticojejunostomy shares pathological similarities with non-obstructive afferent loop syndrome (26). Therefore, duodenoscope-assisted or double-balloon enteroscopy (DBE)-guided deployment of AR-SEMS at the anastomosis may represent a potential therapeutic option for this patient’s recurrent cholangitis.

The fatal outcome of sepsis in this case indicates that conservative management (i.e., lifelong rotating antibiotics) may not represent a sustainable or safe strategy for refractory cholangitis caused by sump syndrome. Although revision surgery (such as lengthening of the Roux-en-Y jejunal limb) does not ensure a definitive cure, the literature supports its role in significantly reducing the frequency and severity of cholangitis episodes. Therefore, after thorough evaluation of the surgical risks、quality of life and the long-term risk of sepsis, surgical approach and therapeutic endoscopy are necessary to prevent the worst outcomes.

In conclusion, accurate differentiation between HC and IgG4-SC can reduce unnecessary surgical procedures in IgG4-SC patients. In IgG4-SC patients with elevated IgG4 levels and fever, secondary infection should be considered. Monotherapy with long-term antibiotics for a patient with recurrent episodes of cholangitis after hepaticojejunostomy may lead to fatal consequences. Surgical lengthening of the jejunal limb or endoscopic placement of a Duckbill-type antireflux self-expandable metal stent may be effective therapeutic options for such patients.

## Data Availability

The original contributions presented in the study are included in the article/supplementary material, further inquiries can be directed to the corresponding author.

## Glossary

RYHJ: Roux-en-Y hepaticojejunostomy
IgG4-SC: IgG4-related sclerosing cholangitis
IgG4-RD: IgG4-related disease
CT: Computed tomography
WBC: White blood cell count
CRP: C-reactive protein
ALT: Alanine aminotransferase
AST: Aspartate aminotransferase
ALP: Alkaline phosphatase
γ-GT: γ-glutamyl transferase
AIP: Autoimmune pancreatitis
HC: Hilar cholangiocarcinoma
CCA: Cholangiocarcinoma
PSC: Primary sclerosing cholangitis
ERCP: Endoscopic retrograde cholangiopancreatography
CDD: Choledochoduodenostomy
AR-SEMS: Duckbill-type anti-reflux self-expandable metal stents
PTC: Percutaneous transhepatic cholangiogram
HIDA: Hepatobiliary iminodiacetic acid
99mTc-PMT: 99mTc-N-pyridoxyl-5-methyltryptophan
DBE: Double-balloon enteroscopy
